# Application and evaluation of a virtual patient system based on a multi-branch dynamic decision model in orthopedic graduate medical education

**DOI:** 10.1186/s12909-026-09327-z

**Published:** 2026-04-28

**Authors:** Jie Xu, Deng Li, Danqi Lu, Meiyi Chen, Tanxiao Chen, Hao Sun, Caicong Wu, Yonghong Qing, Yi Wang

**Affiliations:** 1 Department of Orthopedics, Sun Yat-sen Memorial Hospital, Sun Yat-sen University, No.107 Yanjiang Road West, Guangzhou, Guangdong Province 510120 China; 2 School of Life Sciences, Sun Yat-sen University, No. 135 Xingang Xi Road, Guangzhou, Guangdong Province 510080 China; 3 Department of Medical Education, Sun Yat-sen Memorial Hospital, Sun Yat-sen University, No.107 Yanjiang Road West, Guangzhou, Guangdong Province 510120 China

**Keywords:** Education, Medical, Graduate, Student-centred learning, Patient Simulation

## Abstract

**Background:**

Orthopedic Graduate Medical Education (GME) necessitates the cultivation of comprehensive clinical competencies across various subspecialties—including trauma, joint disorders, spinal conditions, and sports medicine—within a constrained training period. Conventional rotation-based training models often result in uneven exposure to infrequent yet high-risk clinical scenarios, thereby limiting the systematic development of integrative clinical reasoning and awareness of long-term patient prognoses. This study aims to design and evaluate an orthopedic generalist virtual patient artificial intelligence system (OrthoSim AI Agent) that employs a multi-branching, multi-outcome, temporally dynamic decision-making framework spanning the entire continuum of patient care, from initial diagnosis to long-term follow-up. The objective is to enhance orthopedic graduate students’ clinical proficiency within standardized educational programs.

**Methods:**

This single-center, prospective study employing a historical control design enrolled 38 orthopedic graduate students. The control group (*n* = 18) completed conventional rotation training, whereas the intervention group (*n* = 20) received supplementary interactive core disease reinforcement training through the OrthoSim AI Agent in addition to the standard rotation curriculum. The primary outcomes evaluated were the overall and domain-specific scores derived from the Mini-Clinical Evaluation Exercise (Mini-CEX). Secondary outcomes included performance scores obtained from the Objective Structured Clinical Examination (OSCE) station focused on complication management, as well as responses to a custom-designed 5-point Likert scale satisfaction questionnaire.

**Results:**

A longitudinal Mini-CEX analysis (T1–T5) showed both groups started similarly, but the intervention group’s performance significantly surpassed the control group from T4 onward (T4: *P* = 0.012; T5: *P* < 0.001). At T5, the intervention group scored higher in clinical judgment, history-taking, and organizational efficiency (all *P* < 0.05), with greater overall score gains. In the OSCE, the intervention group achieved significantly higher total scores (86.95 ± 9.97 vs. 80.15 ± 4.04, *P* = 0.019). Student satisfaction surveys (5-point Likert scale) revealed high ratings for cross-subspecialty integration (4.25 ± 0.72), safe simulation of high-risk complications (4.45 ± 0.83), and enhanced decision-making confidence (4.15 ± 0.81) (all *P* < 0.001). 80% were “Extremely Likely” to recommend the system.

**Conclusion:**

The OrthSim AI Agent-Assisted Training provides an integrated learning model based on multi-branch dynamic decision-making for orthopedic Graduate Medical Education. By simulating the complete clinical causal chain in a safe environment, it effectively promotes cross-subspecialty thinking, high-risk scenario management, and awareness of long-term prognosis.

## Background

Orthopedics comprises a range of highly specialized subspecialties, including trauma, joint surgery, spine, and sports medicine [1–4]. Within the constraints of the three-year Graduate Medical Education (GME) program, orthopedic graduate students have limited exposure to each subspecialty during their clinical rotations. Furthermore, the core cases and complex complications they encounter are often unpredictable and influenced by variables such as patient-specific conditions and surgical indications [5–8]. This is particularly evident in high-risk, low-frequency procedures—such as spinal deformity correction and pelvic tumor resection with reconstruction—where graduate students frequently assume an observational role rather than engaging actively, thereby missing opportunities for hands-on experience and iterative learning [9].

Although traditional bedside teaching and case discussions provide valuable educational experiences, they are insufficient for systematically replicating the full clinical decision-making process and its long-term outcomes, which may unfold over months or even years [10, 11]. For instance, a graduate student involved in a total knee arthroplasty may not witness within a limited timeframe complications such as early implant loosening due to suboptimal positioning or joint stiffness resulting from inadequate rehabilitation protocols. This absence of “delayed feedback” significantly hampers the development of forward-looking clinical reasoning and risk assessment capabilities [12].

To address these challenges, we developed the “Patient Simulation Orthopedic Intelligent Agent” (OrthoSim AI Agent) and incorporated it into a cross-subspecialty educational platform tailored for orthopedic GME. Unlike systems focused solely on trauma orthopedics, this platform encompasses a virtual case repository representing core pathologies across major orthopedic subspecialties. Its principal innovation lies in a multi-branch architecture governed by layered, time-dependent progression, which not only simulates initial diagnostic and therapeutic decisions within each subspecialty but also models the dynamic progression of characteristic short-term complications (e.g., postoperative infection, nerve injury) and long-term sequelae (e.g., implant loosening, adjacent segment degeneration, tumor recurrence). Each decision made by the graduate student—including surgical approach, implant selection, perioperative management, and rehabilitation planning—is systematically recorded and linked to individualized clinical outcomes at subsequent time points. The present study seeks to assess the efficacy of this system in enhancing the comprehensive clinical competencies of orthopedic graduate trainees.

## Methods

### Study participants and design

**Fig. 1:**
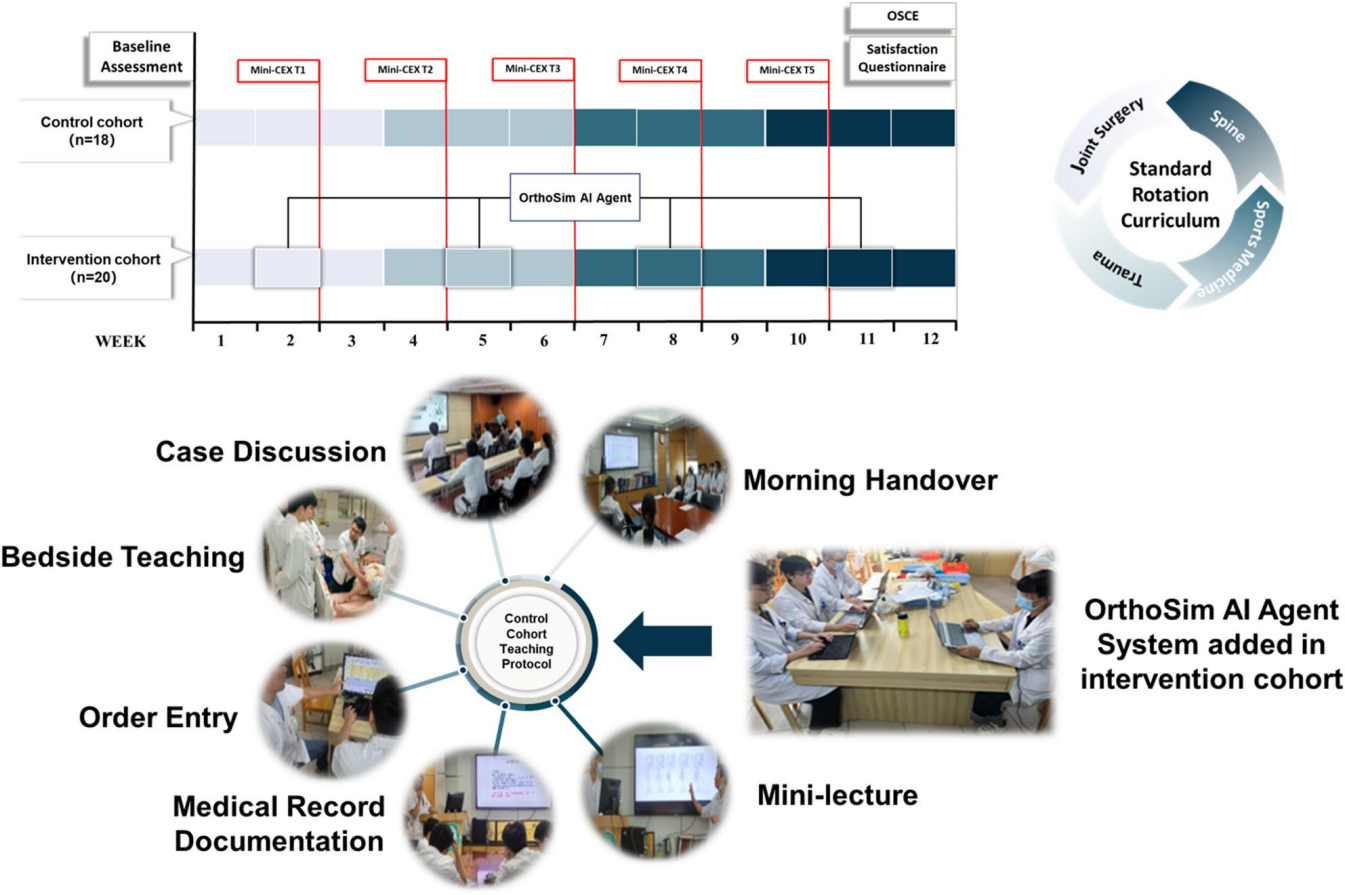
Study Flow Diagram Abbreviations: OrthoSim AI Agent, Patient Simulation Orthopedic Intelligent Agent; Mini-CEX, Mini-Clinical Evaluation Exercise; OSCE, Objective Structured Clinical Examination

This investigation utilized a single-center, prospective historical control study design (Fig. 1). Participants comprised second-year graduate students engaged in standardized graduate student training at the orthopedic specialty training base of our institution. The control group (*n* = 18) included trainees who completed conventional clinical rotation training between September and December 2024. In contrast, the intervention group (*n* = 20) consisted of trainees from the corresponding period in 2025, who in addition to the standard clinical rotation curriculum, participated in an additional four-week training module enhanced by the OrthoSim AI Agent. The overall training duration spanned three months and encompassed four subspecialties: trauma, joint, spine, and sports medicine, with each clinical rotation lasting three weeks. Within the intervention group, one week of OrthoSim specialized training was integrated into the second week of each subspecialty clinical rotation, amounting to a total of four weeks.

To minimize potential confounding attributable to temporal variation between the 2024 (control) and 2025 (intervention) cohorts, the study design incorporated rigorous methodological controls. First, the core clinical curriculum—including rotation structure, learning objectives, and required clinical exposures—remained unchanged across both academic years. Additionally, the roster of supervising faculty and departmental clinical practice guidelines were held constant throughout the study period, thereby mitigating variability in teaching quality or clinical standards.

Second, and critically for internal validity, the OrthoSim AI Agent training module was integrated into the intervention group’s schedule as a direct replacement for an equivalent duration of pre-existing educational activities, specifically designated blocks of self-directed learning and didactic conferences within the standard three-month orthopedic rotation. Consequently, the total amount of structured educational time (“time-on-task”) was identical between groups. This design ensures that any observed differences in outcomes may be attributed to the pedagogical characteristics of the OrthoSim intervention rather than disparities in instructional exposure or effort.

Prior to commencing the training program, all participants completed a standardized baseline competency assessment. Throughout the training period, a consistent evaluation framework was employed by the research team, including Mini-Clinical Evaluation Exercise (Mini-CEX) assessments conducted biweekly, resulting in five longitudinal evaluations. At the conclusion of the training, participants underwent a comprehensive Objective Structured Clinical Examination (OSCE) and completed a structured satisfaction questionnaire.

### Ethics

Ethical exemption for this study was granted by the Medical Education Research Ethics Committee of Sun Yat-sen University Sun Yat-sen Memorial Hospital (Approval No. SYSEC2-BA-060). The project was identified as a quality assurance study aimed at evaluating an educational intervention to improve the implementation of the orthopedic graduate medical education program at Sun Yat-sen Memorial Hospital.

Additionally, to ensure fairness and educational equity, all control group participants were provided with post-study access to the OrthoSim AI Agent immediately following the completion of all assessments, ensuring no trainee was permanently disadvantaged by their group assignment.

### Intervention measures: overview of the OrthoSim AI Agent system

This system is grounded in authentic clinical pathways and has developed an interactive educational platform encompassing the four fundamental domains of orthopedics. It incorporates two principal technologies: a “multi-branch dynamic decision tree” and a “multi-outcome dynamic engine,” thereby establishing a comprehensive, dynamic, and outcome-focused instructional framework. This framework extends from the initial outpatient diagnostic and therapeutic decision-making processes through perioperative management to long-term patient follow-up.

#### Modular content architecture

The OrthoSim AI Agent employs a modular framework, segmenting its system content into four specialized core clinical modules within orthopedic subspecialties:

- Orthopedic Trauma Module: This module addresses both common and complex acute traumatic injuries, including femoral neck and pelvic fractures. It encompasses initial patient history acquisition, identification of characteristic clinical signs, proficiency in imaging interpretation, as well as evaluation of surgical indications and comprehensive postoperative care.
- Joint Surgery Module: Concentrating on degenerative and inflammatory joint pathologies, this module simulates clinical decision-making processes related to conditions such as hip and knee osteoarthritis and rheumatoid arthritis. It covers the selection of prosthesis types, considerations regarding surgical approaches, and perioperative anticoagulation management.
- Spine Surgery Module: This module focuses on prevalent degenerative spinal diseases and structural deformities, providing guidance on diagnosis, treatment planning, perioperative care, long-term follow-up, and rehabilitation protocols.
- Sports Medicine Module: Dedicated to periarticular soft tissue injuries, including anterior and posterior cruciate ligament tears and rotator cuff injuries, this module emphasizes the functional anatomy necessary for accurate diagnosis and differential diagnosis. It also addresses the selection of appropriate reconstruction techniques, rehabilitation strategies, and complication management.

#### Basic system functions

The core interaction mechanism of the system is a multi-branch dynamic decision tree that simulates the nonlinearity and uncertainty of clinical work. OrthoSim AI Agent is built upon a pre-defined, expert-curated “multi-branch dynamic decision tree” that is tightly coupled with a “multi-outcome dynamic engine.” This architecture is grounded in authentic clinical pathways and a comprehensive knowledge base of orthopedic conditions, complications, and management principles. All possible decision nodes, patient responses, and clinical outcomes (both short-term and long-term) are scripted by a team of senior orthopedic clinicians and medical educators. This design ensures complete control over the clinical accuracy and safety of all information presented to the learners, as there is no real-time generation of medical content. The system’s intelligence lies in its complex, non-linear structure and its ability to dynamically link a trainee’s specific decisions to pre-programmed, realistic clinical consequences, thereby simulating the “decision-outcome-feedback” loop without the risks associated with uncontrolled generative models.

At each key node, students actively make judgments:

- Initial Assessment Phase: The system presents the virtual patient’s chief complaint, vital signs, and diagnostic images (such as X-rays and MRIs) but does not provide a diagnosis. Students are required to independently integrate the medical history, perform physical examination reasoning, and interpret imaging.
- Treatment Planning: This is the core decision-making layer. Students must develop a personalized plan by considering factors such as the patient’s age, comorbidities, and imaging characteristics. For example, in an elderly patient with a femoral neck fracture, they must choose between internal fixation and joint replacement; if replacement is chosen, they must further decide between hemiarthroplasty or total hip arthroplasty and specify details such as prosthesis type and fixation method.
- Extended Management Phase: The decision tree continues to extend into the perioperative period (e.g., blood glucose control, deep vein thrombosis prevention), postoperative rehabilitation, and long-term follow-up. At each stage, students are required to input specific management instructions, and the system advances the case accordingly.

#### Multi-outcome dynamic engine

The OrthoSim AI Agent’s multi-outcome dynamic engine links each decision to potential clinical consequences in real time, across two dimensions: short-term and long-term.

- Short-Term Complication Simulation: The system incorporates a built-in clinical risk knowledge base that can immediately trigger corresponding events based on gaps in the treatment plan. For example, if the order for “right lower limb skin traction” is not issued, the system will simulate vascular and nerve injury caused by fracture displacement within 24 h; if blood pressure monitoring is neglected, hidden bleeding may lead to shock; if pressure ulcer care is not arranged, skin breakdown and infection may develop after several days. This design allows students to experience the “cost of negligence” in a risk-free environment.
- Long-Term Complication Simulation: The engine can also track the long-term impact of decisions. For instance, if the prosthesis is poorly positioned during joint replacement, the system simulates “joint dislocation” at follow-up checkpoints; after spinal fusion surgery, it may progress to “adjacent segment degeneration”; if graft tension is improper in anterior cruciate ligament reconstruction, it manifests as “graft failure” and joint instability.

#### Case illustration

To elucidate the system’s functional framework, an elderly patient presenting with a femoral neck fracture is utilized as a representative example. The student is initially introduced to an 82-year-old female patient who reports right hip pain and restricted mobility subsequent to a fall. Radiographic images are provided, and the student is tasked with independently identifying the fracture line, followed by establishing a diagnosis and classification.

Upon accessing the treatment planning module, the student must integrate clinical considerations such as advanced age, osteoporosis, and the patient’s activity requirements to determine the appropriateness of total hip arthroplasty and select a suitable prosthesis. Subsequently, the student formulates perioperative orders, which include directives such as “right lower limb skin traction,” “blood pressure monitoring every 4 hours,” “use of an air mattress combined with scheduled repositioning to prevent pressure ulcers,” and “administration of low molecular weight heparin for anticoagulation.”

The system then engages a dynamic simulation engine that responds to the student’s clinical decisions as follows:

- Omission of “traction” results in the simulation of fracture displacement accompanied by local hematoma formation on the following day;
- Failure to order “blood pressure monitoring” leads to the progression of occult bleeding culminating in hypovolemic shock;
- Neglecting “pressure ulcer prevention” results in the development of a stage II sacrococcygeal pressure ulcer by day five, further complicated by cellulitis.

Moreover, during the three-month postoperative follow-up, the system simulates additional complications based on intraoperative and postoperative management: an excessively large prosthesis anteversion angle triggers recurrent dislocation, while inadequate anticoagulation therapy leads to the simulation of deep vein thrombosis in the ipsilateral lower limb.

### Evaluation metrics and instruments

#### Primary outcome

The Mini-Clinical Evaluation Exercise (Mini-CEX) will serve as the primary assessment instrument. Two senior orthopedic physicians, each with more than 20 years of clinical experience, will independently evaluate graduate students’ clinical consultations with real patients.

#### Secondary outcomes

Objective Structured Clinical Examination (OSCE): A dedicated “Complication Management Station” will be implemented to assess graduate students’ competence in managing acute complications using simulated patient scenarios, including postoperative fever and lower limb weakness.

To ensure the validity of the summative assessment and mitigate the risk of a “teaching-to-the-test” effect, a strict protocol was established to maintain the independence of the OSCE content from the OrthoSim AI Agent training modules. The OSCE station for “Complication Management” was developed by a committee of senior clinical educators who were not involved in the design or content creation of the OrthoSim system. While both the training and the assessment targeted core competencies in managing high-risk orthopedic complications, the specific clinical scenarios, patient presentations, and required critical actions for the OSCE were deliberately distinct from the virtual cases used in the intervention.

#### Student satisfaction survey

A structured questionnaire (Table 1) will be administered to evaluate the system’s effectiveness in promoting cross-subspecialty knowledge integration, increasing exposure to high-risk clinical cases, and strengthening confidence in clinical decision-making.

**Table 1:** 5-point Likert scale satisfaction questionnaie

Section	Question
Basic Information	1. Which orthopaedic subspecialties have you rotated through? (Please check all that apply) □ Trauma Orthopaedics □ Joint Replacement/Arthroplasty □ Spine Surgery □ Sports Medicine □ Orthopaedic Oncology □ Paediatric Orthopaedics □ Other: _______________
Core Functional Value	2. How would you rate the effectiveness of the OrthoSim AI Agent system in helping you understand the connections between different orthopaedic subspecialties (e.g., trauma, joints, spine)? (1 = Very Poor, 5 = Excellent) 3. How would you rate the value of the system in enabling you to safely encounter and manage high-risk, low-frequency complex complications (e.g., periprosthetic joint infection, postoperative cerebrospinal fluid leak after spine surgery)? (1 = Very Poor, 5 = Excellent) 4. How would you rate the system’s effectiveness in enhancing your confidence in making evidence-based, sound clinical decisions when managing complex patients? (1 = Very Poor, 5 = Excellent) 5. Which aspects of the OrthoSim AI Agent system have been most beneficial to your learning? (Please check all that apply) □ Facilitating integration of knowledge across subspecialties (e.g., trauma, joints, spine) □ Safely simulating high-risk, low-frequency complications □ Providing a “safe-to-fail” environment that reduces clinical anxiety □ Reinforcing prospective thinking about long-term consequences of treatment decisions □ Cultivating a “holistic orthopaedic perspective” and multidisciplinary reasoning □ Deepening understanding of trade-offs in surgical decision-making (e.g., hemiarthroplasty vs. total hip arthroplasty)
Benefits	6. Do you believe the OrthoSim AI Agent system addresses a gap in current orthopaedic Graduate Medical Education (GME)? If yes, please specify how (e.g., compensating for limited exposure to X cases during Y subspecialty clinical rotation).
Limitations	7. Did you encounter any scenarios or elements in the system that were confusing, inconsistent with real-world clinical practice, or in need of improvement? □ Yes □ No 8. If you answered “Yes” to the previous question, please describe the issue(s) in detail. 9. What improvements would you suggest for future versions of the OrthoSim AI Agent system? (e.g., add cases from specific subspecialties, enhance certain features, etc.)
Comprehensive Evaluation	10. Overall, how would you rate the OrthoSim AI Agent system as a tool for orthopaedic Graduate Medical Education? (1 = Very Poor, 5 = Excellent) 11. How likely are you to recommend the OrthoSim AI Agent system to fellow graduate students? (1 = Extremely Unlikely, 5 = Extremely Likely) 12. Any additional comments or insights regarding the OrthoSim AI Agent system?

### Statistical analysis

All statistical analyses were conducted using R software, version 4.5.2. A significance threshold of *P* < 0.05 was applied throughout. Variables were reported as means ± standard deviations (SD) and analyzed via Wilcoxon rank sum test. For the evaluation of the platform by students, results were expressed as means accompanied by 95% confidence intervals (95% CI). To assess whether the ratings significantly exceeded a neutral reference value of 3, a one-sample Wilcoxon signed-rank test was performed. Inter-rater reliability for Mini-CEX assessments was quantified using two-way random-effects single-measure ICCs (ICC 2,1) with absolute agreement, based on the mean ratings of two independent assessors. ICC values were interpreted as: <0.50 (poor), 0.50–0.75 (moderate), 0.75–0.90 (good), and > 0.90 (excellent) reliability.

## Results

### Baseline characteristics

This study involved 38 postgraduate students enrolled in an integrated orthopedic residency program, with 20 individuals allocated to the intervention group and 18 to the control group. A comparative analysis of baseline characteristics indicated that all participants in both groups were male. Furthermore, no statistically significant differences were observed between the groups in terms of age (26.87 ± 2.51 vs. 26.83 ± 2.62 years, *P* = 0.958) or pre-department comprehensive assessment scores (74.90 ± 7.78 vs. 70.60 ± 5.49, *P* = 0.582), confirming that the groups were well matched at baseline.

### Mini-CEX formative assessment and OSCE summative assessment

#### Findings from the Mini-CEX formative assessment

**Fig. 2:**
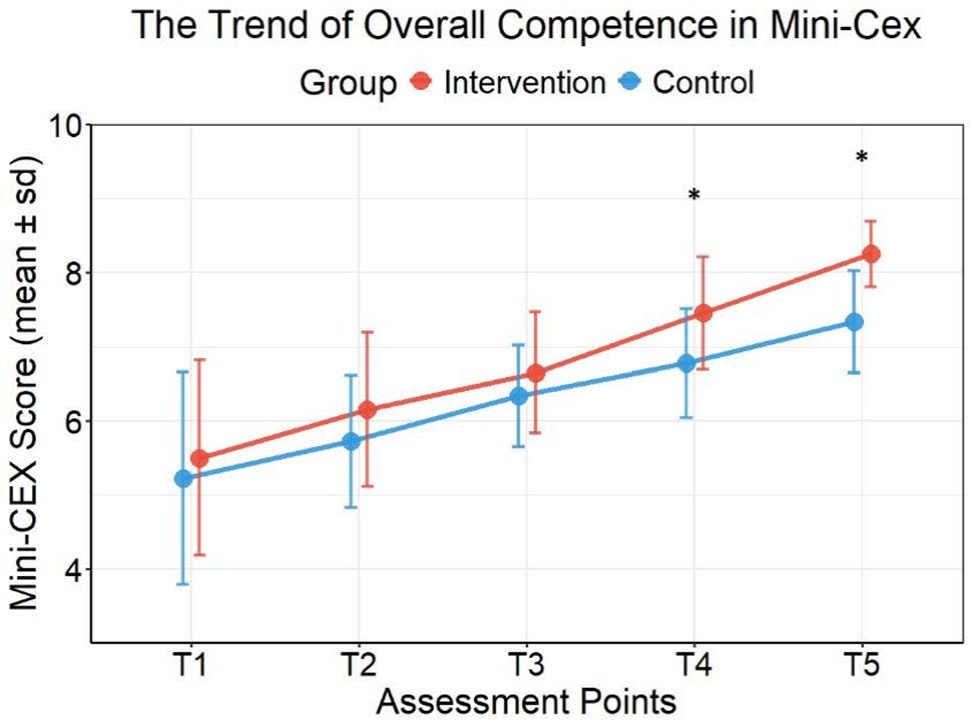
The Trend of Overall Competence in Mini-Cex (* *P* < 0.05)

During the three-month clinical rotation, all participants completed five Mini-CEX formative assessments (T1 to T5) according to the instructional schedule, facilitating ongoing evaluation of clinical competency development. A longitudinal analysis of overall competence scores (Fig. 2) demonstrated distinct patterns between the two groups: the intervention group (*N* = 20) exhibited a steady increase in Mini-CEX performance, with scores rising from 5.50 ± 1.32 at T1 to 8.25 ± 0.44 at T5. Conversely, the control group (*N* = 18) displayed more limited improvement, with overall competence scores remaining relatively stable, ranging from 5.22 ± 1.44 at T1 to 7.33 ± 0.69 at T5.

Further statistical analysis results (Table 1, Fig. 2) showed that in the early assessment phases from T1 to T3, there was no statistically significant difference in the overall competence scores between the two groups (T1: *P* = 0.556; T2: *P* = 0.167; T3: *P* = 0.274), indicating that the clinical ability bases of the two groups were at a similar level at this stage. However, starting from the T4 assessment node, the scores of the intervention group began to diverge from those of the control group: at T4, the overall competence score of the intervention group was 7.45 ± 0.76, which was significantly higher than the control group’s 6.78 ± 0.73 (*P* = 0.012). By the final T5 assessment phase, this gap further widened, and the comparison between the intervention group’s score (8.25 ± 0.44) and the control group’s score (7.33 ± 0.69) showed *P* < 0.001, suggesting that a statistically significant difference in comprehensive clinical ability had formed between the two groups.

**Fig. 3:**
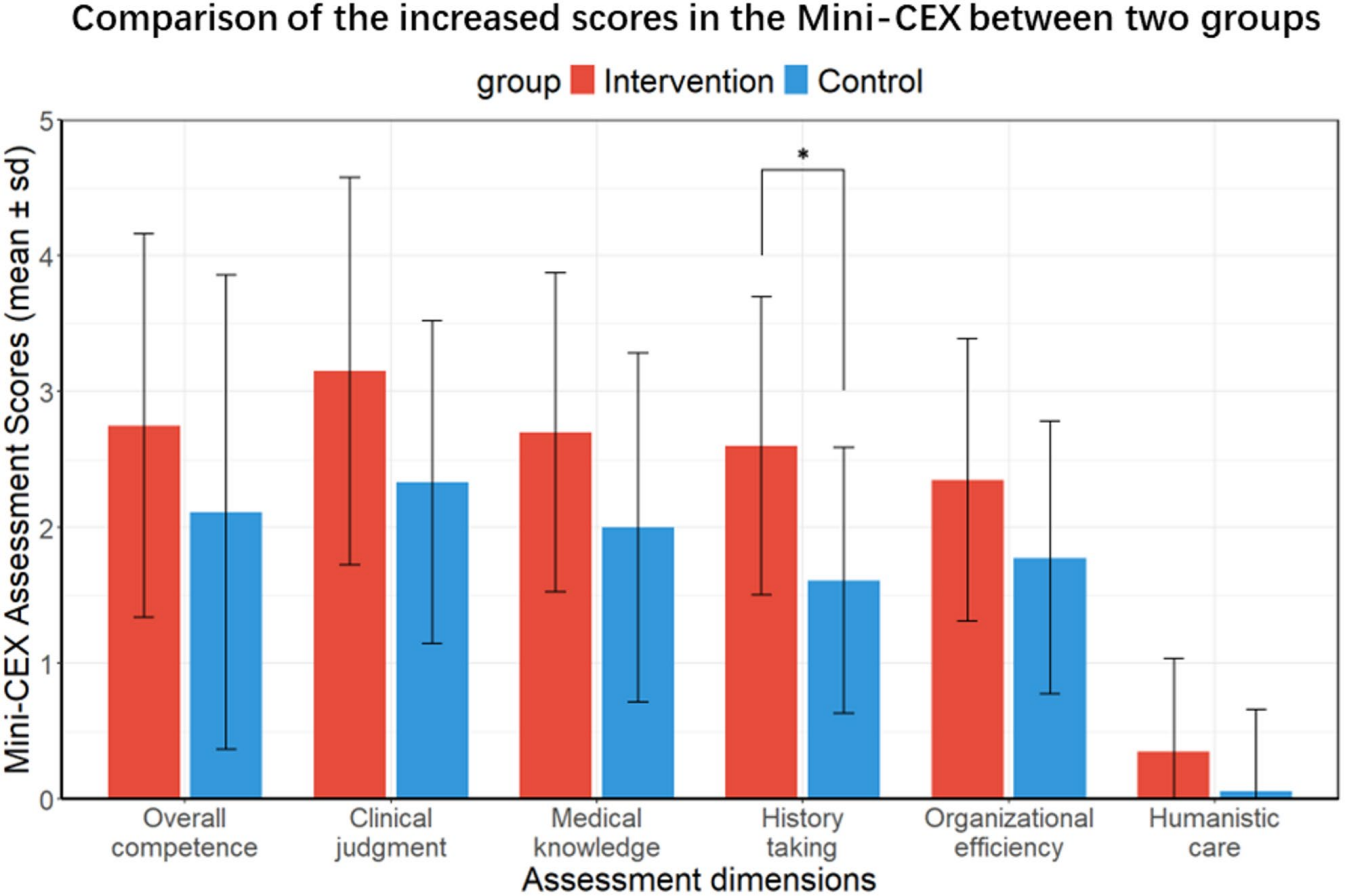
Comparison of the increased scores in the Mini-CEX between two groups (* *P* < 0.05)

Meanwhile, from the perspective of inter-group comparison of multi-dimensional abilities (Table 2), at the T5 phase, the intervention group’s scores in the 3 core assessment dimensions—clinical judgment, history-taking skills, and organizational efficiency management—were significantly higher than the corresponding scores of the control group (*P* < 0.05). Notably, from the perspective of score increments, the intervention group showed a more prominent improvement in all assessment dimensions (Fig. 3): taking the history-taking dimension as an example, the intervention group’s score increased from 5.35 ± 0.93 at T1 to 7.95 ± 0.60 at T5, with a score increase of 2.60 points; in contrast, the control group only increased from 5.61 ± 0.85 at T1 to 7.22 ± 0.43 at T5 in this dimension, with an increase of only 1.61 points. These results collectively indicate that the intervention implemented in this study not only effectively improved the trainees’ comprehensive clinical competence but also demonstrated a more significant promoting effect on multi-dimensional ability development, enabling a more comprehensive advancement of the growth and improvement of trainees’ clinical skills.

**Table 2:** Comparison of Mini-CEX Median Scores Between Intervention and Control Groups Across Five Assessment Points

Assess points	Mini-CEX dimension	Intervention *N* = 20	Control *N* = 18	*p*-value
T1	Overall competence, Mean ± SD	5.50 ± 1.32	5.22 ± 1.44	0.556
	Clinical judgment, Mean ± SD	4.75 ± 1.16	5.06 ± 1.21	0.426
	Medical knowledge, Mean ± SD	5.15 ± 0.93	5.50 ± 0.92	0.245
	History taking, Mean ± SD	5.35 ± 0.93	5.61 ± 0.85	0.392
	Organizational efficiency, Mean ± SD	5.55 ± 0.89	5.44 ± 0.86	0.720
	Humanistic care, Mean ± SD	7.90 ± 0.72	7.94 ± 1.00	0.690
T2	Overall competence, Mean ± SD	6.15 ± 1.04	5.72 ± 0.89	0.167
	Clinical judgment, Mean ± SD	5.85 ± 0.93	5.83 ± 0.92	0.975
	Medical knowledge, Mean ± SD	6.35 ± 1.50	5.56 ± 0.98	0.057
	History taking, Mean ± SD	6.45 ± 1.57	5.67 ± 1.03	0.107
	Organizational efficiency, Mean ± SD	6.15 ± 1.09	5.39 ± 0.70	0.009
	Humanistic care, Mean ± SD	8.05 ± 0.83	8.17 ± 0.71	0.684
T3	Overall competence, Mean ± SD	6.65 ± 0.81	6.33 ± 0.69	0.274
	Clinical judgment, Mean ± SD	5.90 ± 0.79	5.72 ± 0.96	0.345
	Medical knowledge, Mean ± SD	6.95 ± 0.69	6.00 ± 1.08	0.002
	History taking, Mean ± SD	6.30 ± 0.92	5.72 ± 1.07	0.066
	Organizational efficiency, Mean ± SD	7.00 ± 0.56	6.17 ± 1.10	0.001
	Humanistic care, Mean ± SD	7.95 ± 0.83	7.83 ± 0.92	0.764
T4	Overall competence, Mean ± SD	7.45 ± 0.76	6.78 ± 0.73	0.012
	Clinical judgment, Mean ± SD	7.25 ± 0.79	6.17 ± 0.86	< 0.001
	Medical knowledge, Mean ± SD	7.30 ± 0.80	6.17 ± 0.79	< 0.001
	History taking, Mean ± SD	7.35 ± 0.75	6.28 ± 0.67	< 0.001
	Organizational efficiency, Mean ± SD	7.50 ± 0.69	6.17 ± 0.99	< 0.001
	Humanistic care, Mean ± SD	7.80 ± 0.89	7.83 ± 0.92	0.887
T5	Overall competence, Mean ± SD	8.25 ± 0.44	7.33 ± 0.69	< 0.001
	Clinical judgment, Mean ± SD	7.90 ± 0.64	7.39 ± 0.70	0.012
	Medical knowledge, Mean ± SD	7.85 ± 0.81	7.50 ± 0.71	0.092
	History taking, Mean ± SD	7.95 ± 0.60	7.22 ± 0.43	< 0.001
	Organizational efficiency, Mean ± SD	7.90 ± 0.55	7.22 ± 0.65	< 0.001
	Humanistic care, Mean ± SD	8.25 ± 0.72	8.00 ± 0.69	0.271

#### Inter-rater reliability between expert assessors

To ensure the reliability of the Mini-CEX assessments, two independent senior orthopedic physicians evaluated each encounter. Inter-rater reliability was analyzed using two-way random-effects single-measure ICCs (ICC 2,1) with absolute agreement (Table 3).

**Table 3:** Inter-rater Reliability (ICC) of Mini-CEX Dimensions Across Five Assessment Points

	Overall Competence	Clinical Judgment	Medical Knowledge	History Taking	Organizational Efficiency	Humanistic Care
T1	0.84 (0.71–0.92)	0.82 (0.68–0.91)	0.86 (0.74–0.93)	0.79 (0.64–0.89)	0.81 (0.67–0.90)	0.77 (0.61–0.88)
T2	0.87 (0.76–0.94)	0.85 (0.73–0.93)	0.89 (0.79–0.95)	0.82 (0.69–0.91)	0.84 (0.72–0.92)	0.80 (0.66–0.90)
T3	0.88 (0.78–0.94)	0.87 (0.76–0.94)	0.91 (0.82–0.96)	0.84 (0.72–0.92)	0.86 (0.75–0.93)	0.82 (0.69–0.91)
T4	0.90 (0.81–0.95)	0.89 (0.79–0.95)	0.92 (0.84–0.96)	0.86 (0.75–0.93)	0.88 (0.78–0.94)	0.84 (0.72–0.92)
T5	0.92 (0.85–0.96)	0.91 (0.83–0.96)	0.94 (0.87–0.97)	0.88 (0.78–0.94)	0.90 (0.81–0.95)	0.86 (0.75–0.93)

Across all five assessment timepoints (T1–T5), inter-rater reliability for the Mini-CEX evaluations ranged from 0.77 to 0.94, indicating good to excellent consistency between the two expert raters. Notably, the domain of Medical Knowledge exhibited the highest inter-rater reliability (ICC: 0.86–0.94), likely reflecting the relatively objective and criterion-based nature of factual clinical knowledge assessment. In contrast, Humanistic Care demonstrated comparatively lower—but still acceptable—agreement (ICC: 0.77–0.86), which aligns with the inherently subjective dimensions of evaluating interpersonal and communication skills.

#### OSCE final evaluation outcomes

Upon completion of the clinical rotation, all students underwent the standardized OSCE final examination. The intervention group achieved an overall OSCE score of 86.95 ± 9.97, which was significantly higher than the control group’s total score of 80.15 ± 4.04 (*P* = 0.019). Within the assessment stations, the “Complication Emergency Management” station was designated as a key competency evaluation, emphasizing the students’ proficiency in identifying and managing acute postoperative complications in orthopedic practice. The intervention group attained a mean score of 90.15 ± 7.82 at this station, which was significantly higher than the control group’s mean score of 84.93 ± 7.28 (*P* = 0.042). Structured feedback provided by standardized examiners revealed that students in the intervention group exhibited a more systematic approach to differential diagnosis, clearer clinical management reasoning, and enhanced evidence-based decision-making capabilities when confronted with complex and atypical clinical cases. These results align with the competency enhancements observed in the Mini-CEX evaluations.

### Enhancement of cross-subspecialty integration competence

Qualitative analysis of interview data reveals that participants in the intervention group generally perceived the system as effective in fostering the integration of clinical reasoning across various orthopedic subspecialties. Through repeated engagement with core cases encompassing multiple domains—including trauma, joint disorders, spinal conditions, and sports medicine—within a simulated virtual environment, students developed a comprehensive “orthopedic holistic perspective” that transcends the confines of individual subspecialties. Notably, during diagnostic evaluation and therapeutic decision-making, participants actively identified and synthesized potential interconnections among diverse disease spectra. For instance, in managing geriatric hip fracture cases, they anticipated implications for long-term joint degeneration; similarly, when planning ligament reconstruction procedures in younger patients, considerations of graft selection and subsequent arthritis risk were integrated into their clinical reasoning. This integrative cognitive framework is challenging to systematically cultivate through conventional departmental clinical rotation models.

### Student satisfaction and perceived value

To thoroughly assess students’ subjective experiences and acceptance of the OrthoSim AI Agent system, this study utilized a 5-point Likert scale questionnaire encompassing sections such as Basic Information, Core Functional Value, Benefits, Limitations, and Overall Evaluation. The findings indicated that participants in the intervention group consistently provided high ratings across all primary dimensions of the system, with mean scores ranging from 4.15 to 4.65, all statistically significant at *P* < 0.001, and notably exceeding the neutral midpoint of 3 on the scale.

**Fig. 4:**
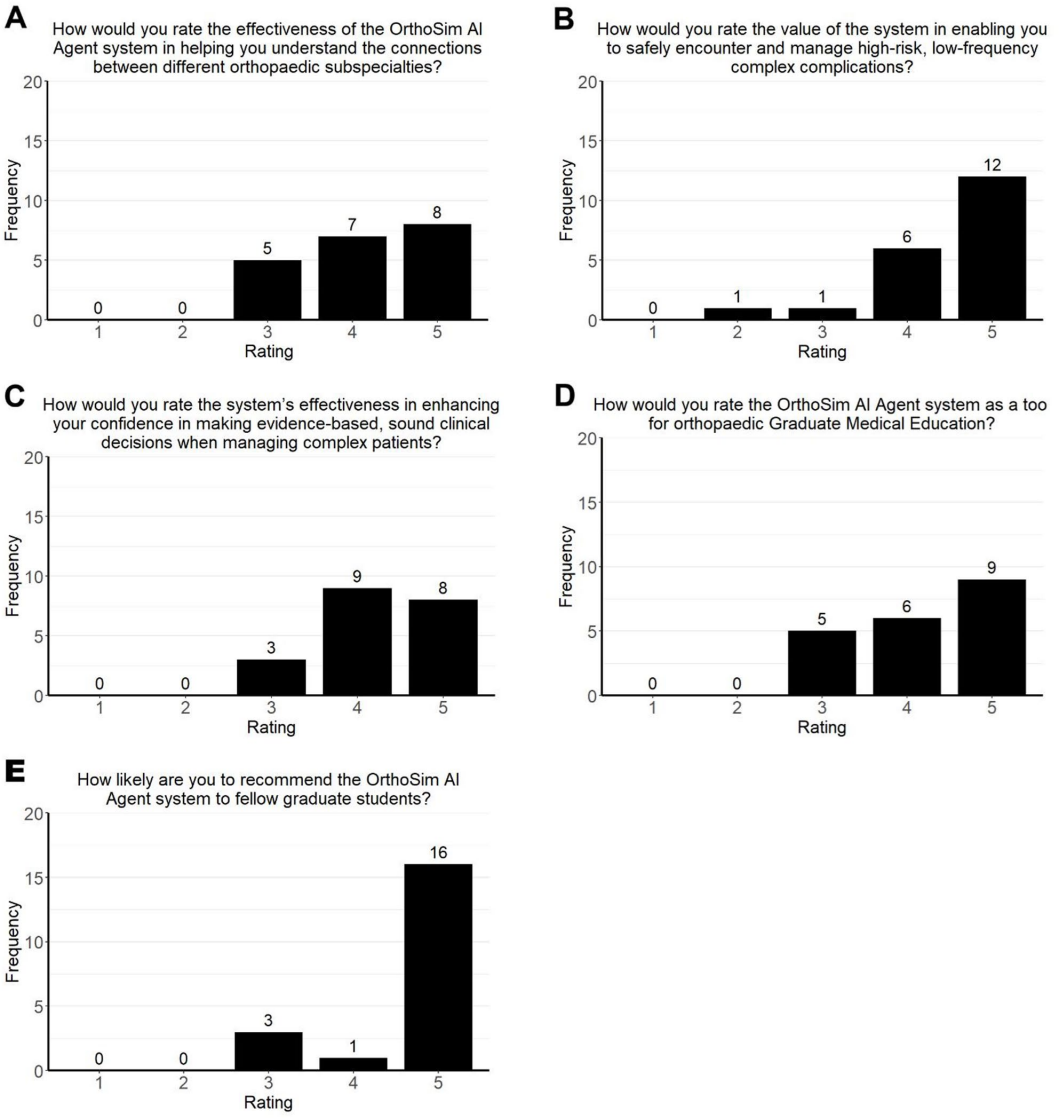
Students’ Ratings of the OrthoSim AI Agent System **A** Effectiveness in helping understand connections between orthopaedic subspecialties; **B** Value in enabling safe encounter and management of high-risk, low-frequency complex complications; **C** Effectiveness in enhancing confidence for evidence-based, sound clinical decisions when managing complex patients; **D** Perceived utility as a tool for orthopaedic graduate medical education; **E** Likelihood of recommending the system to fellow graduate students

Specifically, regarding the integration of cross-subspecialty knowledge (Fig. 4A), the item “The system helps understand connections between orthopaedic subspecialties” received a mean score of 4.25 ± 0.72. Concerning exposure to high-risk, low-frequency cases (Fig. 4B), the statement “The system enables safe management of complex complications” was rated at 4.45 ± 0.83. In relation to the enhancement of clinical decision-making confidence (Fig. 4C), the item “The system boosts confidence in evidence-based decision-making” achieved a mean score of 4.15 ± 0.81.

**Fig. 5:**
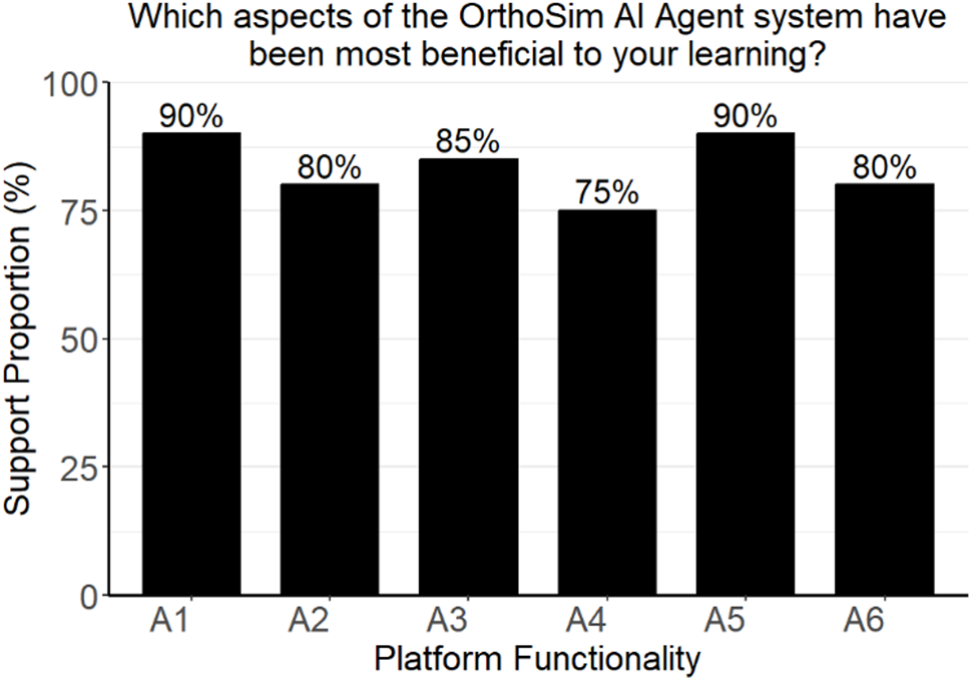
Students’ Perceived Key Benefits of the OrthoSim AI Agent System. A1: Facilitating integration of knowledge across subspecialties (e.g., trauma, joints, spine). A2: Safely simulating high-risk, low-frequency complications. A3: Providing a “safe-to-fail” environment that reduces clinical anxiety. A4: Reinforcing prospective thinking about long-term consequences of treatment decisions. A5: Cultivating a “holistic orthopaedic perspective” and multidisciplinary reasoning. A6: Deepening understanding of trade-offs in surgical decision-making (e.g., hemiarthroplasty vs. total hip arthroplasty)

The benefit dimension survey presented in Fig. 5 further corroborates these findings: 90% of participants acknowledged the system’s effectiveness in “Facilitating cross-subspecialty knowledge integration,” 80% concurred that it “Enables safe simulation of high-risk complications,” 85% affirmed that it “Provides a safe-to-fail environment to reduce clinical anxiety,” 90% recognized its role in “Cultivating a holistic orthopaedic perspective,” and 75% indicated that it “Reinforces prospective thinking in treatment decisions.” These results clearly underscore the system’s fundamental contributions to knowledge integration, risk simulation, psychological support, and related domains.

Regarding the comprehensive evaluation (Fig. 4D-E), the item assessing “The system’s value as a GME training tool” received a mean score of 4.2 ± 0.83, with 80% of students expressing that they were “Extremely Likely” to recommend the system to their peers. Collectively, the feedback emphasizes the system’s efficacy in fostering competency development and its practical utility. Both quantitative metrics and qualitative responses suggest that the OrthoSim AI Agent’s repeatable, zero-risk immersive simulation environment not only enhances students’ comprehension of complex clinical scenarios but also substantially improves their professional confidence and decision-making skills when confronting real-world clinical challenges.

## Discussion

This research validates that the OrthoSim AI Agent virtual patient system effectively supports intelligent instruction throughout the comprehensive domain of orthopedics and exhibits substantial educational benefits across various evaluative criteria. By employing a multi-branch dynamic decision model architecture, the system not only mitigates the inherent limitations associated with conventional rotation-based teaching methods but also significantly contributes to fostering integration across subspecialties, improving competencies in managing high-risk clinical situations, augmenting understanding of long-term prognostic outcomes, and promoting educational equity.

### Interdisciplinary integration

This system effectively addresses the common challenge of “disciplinary fragmentation” often encountered in traditional subspecialty clinical rotations. Empirical evidence demonstrates that students participating in the intervention group achieved significantly higher scores than the control group on the “clinical judgment” component of the Mini-CEX assessment. Furthermore, they rated the “interdisciplinary integration” aspect highly (4.25 ± 0.72) in the satisfaction survey. These findings suggest that by consistently engaging with dynamic cases spanning various subspecialties—such as trauma, joints, spine, and sports medicine—within a unified platform, students progressively develop a holistic orthopedic perspective that goes beyond a single specialty focus. These results are further corroborated by structured feedback from standardized examiners, who noted that intervention-group students exhibited a more systematic approach to differential diagnosis, clearer clinical reasoning, and stronger evidence-based decision-making when managing complex or atypical cases—competencies directly aligned with the enhancements observed in Mini-CEX evaluations [13].

The robust inter-rater reliability (ICC > 0.80 across all domains) ensured that the observed improvements in the intervention group reflected genuine competency development rather than measurement error. Notably, the high consistency in ‘Clinical Judgment’ assessments (ICC: 0.82–0.91) validated the system’s effectiveness in cultivating cross-subspecialty reasoning capabilities.

### Safe practice of high-risk scenarios

The OrthoSim AI Assistant offers students a secure and consistent platform to simulate high-risk, infrequent clinical events. Participants using this system demonstrated significantly better performance at the OSCE Final Evaluation compared to the control group (90.15 ± 7.82 vs. 84.93 ± 7.28, *P* < 0.05), and received excellent ratings on the “High-Risk Scenario Experience” metric (4.45 ± 0.83), confirming the system’s effectiveness. In real-world clinical practice, serious complications—such as cerebrospinal fluid leaks following spinal deformity surgery or deep prosthetic infections after joint replacement—are rare and unpredictable. This often limits students to passive observation or causes them to miss critical learning opportunities altogether. The OrthoSim system, with its dynamic complication engine, creates a zero-risk simulated environment where students can repeatedly practice, experiment, and immediately see both the short- and long-term outcomes of their decisions, including choices related to anticoagulation regimens and wound management strategies. This approach systematically enhances students’ skills in early recognition, differential diagnosis, and multidisciplinary collaborative management under pressure. The structured and repeatable depth of learning provided by this system significantly surpasses traditional clinical rotations, which depend largely on chance case exposure [14, 15].

### Long-term perspective

This system greatly improves students’ comprehension of the long-term effects of treatment, promoting the growth of professional responsibility and compassionate care. The positive feedback on the “Decision Confidence” measure (4.15 ± 0.81) indicates a meaningful transformation in their clinical reasoning—from concentrating solely on the technical aspects of “how to perform surgery” to thoughtfully considering the significant implications of “why this decision” is made, including its impact on patients’ long-term function, quality of life, and risk of reoperation. Within the virtual environment, students directly experience “delayed consequences,” such as deep vein thrombosis due to inadequate perioperative anticoagulation or joint stiffness from insufficient rehabilitation guidance, thereby clearly linking decisions to outcomes. This reflective, experiential learning process profoundly shapes their evidence-based, cautious, and patient-centered clinical approach. Critically, longitudinal Mini-CEX data reveal that while both groups started at comparable baseline levels, the intervention group’s performance diverged significantly from T4 onward, culminating in a markedly higher overall competence score (7.45 ± 0.76 vs. 6.78 ± 0.73, *P* = 0.012). These results suggest that the intervention—grounded in the multi-branch dynamic decision-making model—effectively accelerated the development of clinical competencies, particularly in the later stages of training, leading to statistically and educationally significant advantages by the final assessment.

### Fairness and scalability

The introduction of the OrthSim AI Agent-Assisted Training has significantly improved the fairness and scalability of educational resources. It ensures that all students have equal access to valuable, complex cases, thereby eliminating skill gaps that often arise from uneven case distribution in traditional teaching methods. Additionally, the system is highly adaptable, continuously expanding its case library and decision pathways to reflect the latest clinical guidelines and advancements in the field. This approach guarantees that the educational content remains current and aligned with best practices [16].

### Limitations

Despite its promising outcomes, this study has several limitations. First, it was conducted at a single institution, which may limit the generalizability of findings to other training environments with different curricula or resource levels. Second, the use of a historical control group, while pragmatic, introduces potential confounding from temporal changes in clinical practice or assessment standards between 2024 and 2025. Third, the system primarily targets cognitive and decision-making domains; as evidenced by the lack of difference in the “communication and humanistic care” dimension of Mini-CEX, it does not substitute for real patient interactions in developing interpersonal skills. Finally, long-term retention of learned competencies and transfer to actual clinical performance beyond the training period were not assessed and warrant investigation in future longitudinal studies.

## Conclusion

The OrthSim AI Agent-Assisted Training provides an integrated learning model based on multi-branch dynamic decision-making for orthopedic Graduate Medical Education. By simulating the complete clinical causal chain in a safe environment, it effectively promotes cross-subspecialty thinking, high-risk scenario management, and awareness of long-term prognosis.

## Data Availability

The datasets generated during this study are not publicly available due to institutional policy but are available from the corresponding author on reasonable request for researchers who meet the criteria for access to confidential data.

## Abbreviations

GME: Graduate Medical Education
OrthoSim AI Agent: Patient Simulation Orthopedic Intelligent Agent
Mini-CEX: Mini-Clinical Evaluation Exercise
OSCE: Objective Structured Clinical Examination
